# Phylogenetic relationship and characterization of the complete mitochondrial genome sequence of *Opsarius caudiocellatus* (Cypriniformes: Danionidae: Chedrinae)

**DOI:** 10.1080/23802359.2022.2151324

**Published:** 2022-12-07

**Authors:** Xiao Jiang Chen, Lin Song, You Wen Cao, Quan Wang

**Affiliations:** College of Fisheries Science and Technology, Jiangsu Agri-animal Husbandry Vocational College, Taizhou, China

**Keywords:** *Opsarius caudiocellatus*, Chedrinae, *Barilius caudiocellatus*, *Barilius barila*, *Opsarius canarensis*

## Abstract

In this paper, we first report the complete mtDNA sequence of *Opsarius caudiocellatus* with the main aim of providing a basis for further researches of this species. Assembly circular mitogenome was 16,534 bp long encoded 13 protein-coding genes, two ribosomal RNA genes, 22 transfer RNA genes, and one control region. The gene nucleotide composition was estimated to be 28.1% A, 25.2% T, 18.6% G, 28.1% C. A maximum-likelihood (ML) phylogenetic tree was reconstructed using the 13 concatenated mitochondrial protein-coding genes of *O. caudiocellatus* and other 19 species of the subfamily Chedrinae. Result of the phylogenetic analysis revealed that *O. caudiocellatus* was well grouped with *Barilius barila.* This study could enrich genetic resources and be helpful to studies on evolution and conservation genetics for *O. caudiocellatus.*

## 1. Introduction

**Figure 1.**
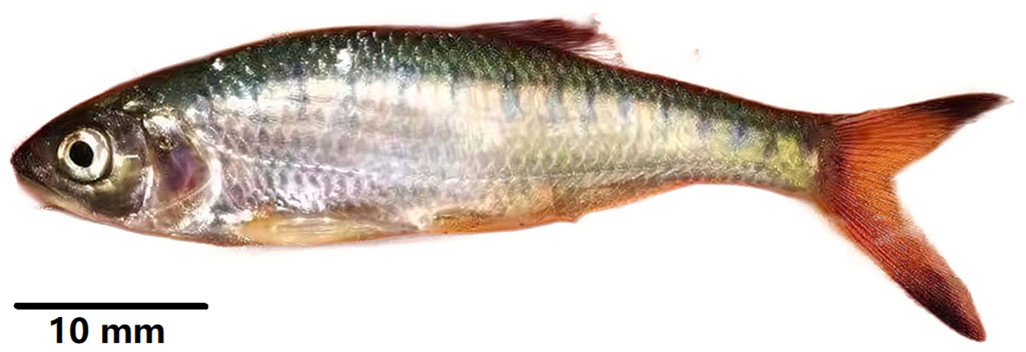
*Opsarius caudiocellatus* was collected from Nujiang River in Lushui County, Yunnan Province of China in May 2021 (photo by Xiao Jiang CHEN).

*Opsarius caudiocellatus* (Chu 1984; Cypriniformes: Danionidae: Chedrinae) is an endemic small freshwater cyprinid fish (∼111 mm), which inhabits medium to fast flowing rivers or mountain streams with rich dissolved oxygen, medium to low slope, the substrate of gravel, cobbles and large pebbles. It is mainly distributed in the Nujiang River and Lancang River in China, and it is also distributed in India, Thailand, Vietnam, and Laos. In the past, *Barilius caudiocellatus* was treated as the synonym name of *O. caudiocellatus*. The main morphological features of *O. caudiocellatus* are similar to those of *Barilius barila* such as dorsal fin iii, 7–8; anal fin ii, 9–11; pectoral fin i, 11–12; ventral fin i, 7, anal fin origin opposite the 4th–7th branching dorsal fin. *O. caudiocellatus* can be distinguished from *Barilius barila* as follows: black spots at the base of the caudal fin for *O. caudiocellatus* not for *B. barila*, and the black area is positioned in the middle of the dorsal fin for *O. caudiocellatus*, while on both sides of *B. barila* (Figure 1) (Chu 1984). The complete mitochondrial genome is regularly used in detailed distribution surveys which are urgently required for this species, and thus could more precisely reveal the taxonomic status (Qin et al. 2019). Nevertheless, up to the present, the complete mitochondrial genome of only four species in the genus *Opsarius* (*O. caudiocellatus*, *O. canarensis*, *O. pulchellus*, and *O. bernatziki*) can be retrieved in CNBI. In this paper, we firstly report the complete mtDNA sequence of *O. caudiocellatus*, with the main aim of providing a basis for further research and conservation of this species.

## 2. Materials and methods

### 2.1. Sample collection and preservation

The specimens were collected by gill nets from Nujiang River in Lushui County, Yunnan Province of China (25°84′75.91″ N, 98°86′69.44″ E) in May 2021, under the permission granted by Jiangsu Agri-animal Husbandry Vocational College (NSF2021ZR14). Research involving laboratory animals followed the ARRIVE guidelines (https://arriveguidelines.org/). The dead fish were selected while the live fish were released into the river. Samples were fixed in 95% ethanol and stored at the Aquatic Science and Technology Institution Herbarium (https://www.jsahvc.edu.cn/, Voucher number ASTIH-21b1108d30, Chen Xiao Jiang, 2007020030@jsahvc.edu.cn). According to the morphological characteristics description of *O. caudiocellatus* by Chu (1984), with tools such as vernier calipers and an anatomical microscope, we identified the species by morphometry and meristematic counts.

### 2.2. Mitochondrial genome sequencing

In this paper, high-throughput sequencing technology was used. Genomic DNA was extracted from a specimen muscle using the Tguide Cell/tissue genomic DNA Extraction Kit (OSR-M401) (Tiangen, Beijing, China). The main experimental steps are as follows: (1) DNA sample quality control; (2) DNA Library Construction; (3) PCR Amplification; (4) Size Selection; (5) Library Quality Check; (6) Library Pooling and Sequencing, the amplified original library was submitted to Illumina HiSeq 4000 Sequencing platform (Illumina, CA, USA).

### 2.3. Assembly, annotation, and analysis

The quality check process was conducted on FastQC Version 0.11.8 (Andrews 2015) to obtain clean data, and the mitogenome was assembled from the clean data (2.46 GB) by MetaSPAdes 3.13.0 (Nurk et al. 2017) with *Barilius malabaricus* MN650735.1 as reference (Prabhu et al. 2020). The resulting circular contig consensus sequence was annotated and verified with MITOS WebServer (http://mitos.bioinf.uni-leipzig.de/index.py) (Bernt et al. 2013). Genome Map was drawn by OGDRAW (https://chlorobox.mpimp-golm.mpg.de/OGDraw.html) (Greiner et al. 2019). MEGA X was used for alignments, analyses, model calculation, and phylogeny reconstruction (Kumar et al. 2018). Maximum-likelihood (ML) phylogenetic tree was reconstructed using the concatenated mitochondrial protein-coding genes of *O. caudiocellatus* and 19 published species. *Rasbora lateristriata* (Kusuma and Kumazawa 2016), and *Rasbora steineri* (Chang et al. 2013) were used as outgroups to root the tree. The best evolutionary model obtained the lowest Bayesian information standard scores (Nei and Kumar 2000).

## 3. Results and discussion

### 3.1. Genomic analysis results

**Figure 2.**
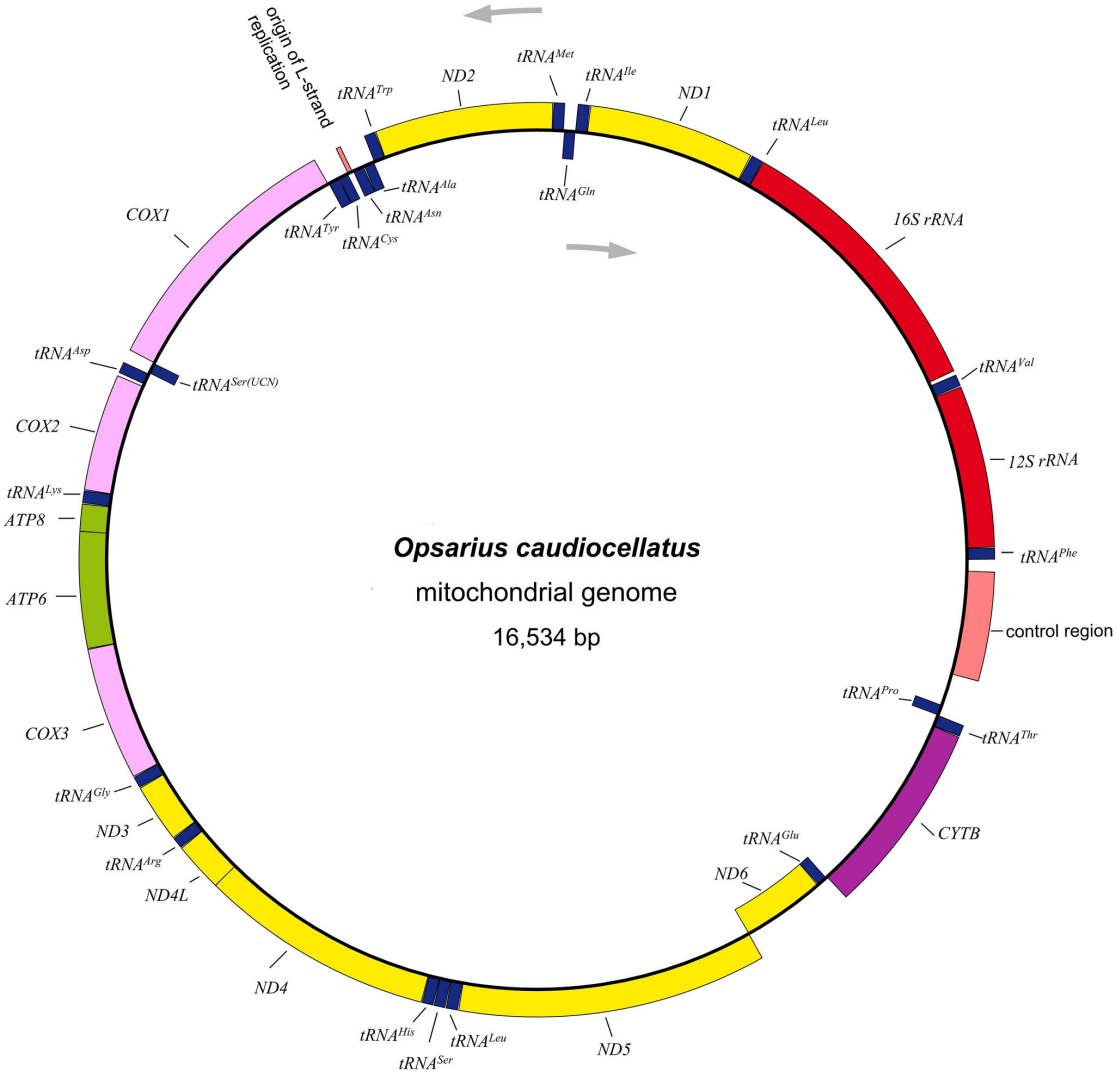
Mitochondrial genome map of *Opsarius caudiocellatus*. The arrows represents direction of transcription, H-strand is located in the outer ring and L-strand is located in the inner ring.

The sequenced mtDNA of *O. caudiocellatus* was 16,534 bp in length, comprised of typical vertebrates’ mitogenome components: 13 protein-coding genes, two ribosomal RNA genes, 22 transfer RNA genes, and one control region (CR or D-loop) (Figure 2). The data was deposited at the GenBank with accession No. OM617729. Like other Chedrinae mitogenomes (Luo et al. 2019; Tan et al. 2020), nine of the thirty-seven genes (*ND6*, *tRNA^Gln^*, *tRNA^Ala^*, *tRNA^Asn^*, *tRNA^Cys^*, *tRNA^Tyr^*, *tRNA^Ser(UCN)^*, *tRNA^Glu^*, and *tRNA^Pro^*) were observed to be encoded on the L-strand (Prabhu et al. 2020; Chen et al. 2022). The overall nucleotides composition for A, T, G, and C was estimated to be 28.1%, 25.2%, 18.6%, and 28.1%, reflecting the relatively higher percentage of AT (53.3%) than GC (46.7%). *CO1* and *ND4L* set the non-canonical GTG as the start codon as the other protein-coding genes initiated with the same ATG. Four types of stop codon usage were found, including typical termination codon TAA (*ND1*, *CO1*, *ATP8*, *ATP6*, *CO3*, *ND3*, *ND4L*, and *ND5*), TAG (*ND2* and *ND6*), and incomplete termination codon T (*CO2* and *CYTB*), TA (*ND4*). As for the protein-coding genes, the longest was *ND5* (1812 bp) while the shortest was *ATP8* (165 bp). Similar to most vertebrate mitogenomes, four pairs of protein-coding genes, namely *ATP8-ATP6*, *ATP6-CO3*, *ND4L-ND4*, and *ND5-ND6*, showed the overlapping which spanned 7, 1, 7,4, respectively (Wang et al. 2019; Yang et al. 2020). 22 tRNAs ranged from 65 bp of cysteine to 76 bp of lysine in length. 12S rRNA (953 bp) and 16S rRNA (1,658 bp) were the two subunits of rRNA, which were positioned between *tRNA^Phe^* and *tRNA^Leu(UUR)^*. The CR spanned 361 bp length and was commonly located between *tRNA^Pro^* and *tRNA^Phe^*.

### 3.2. Phylogenetic analysis

**Figure 3.**
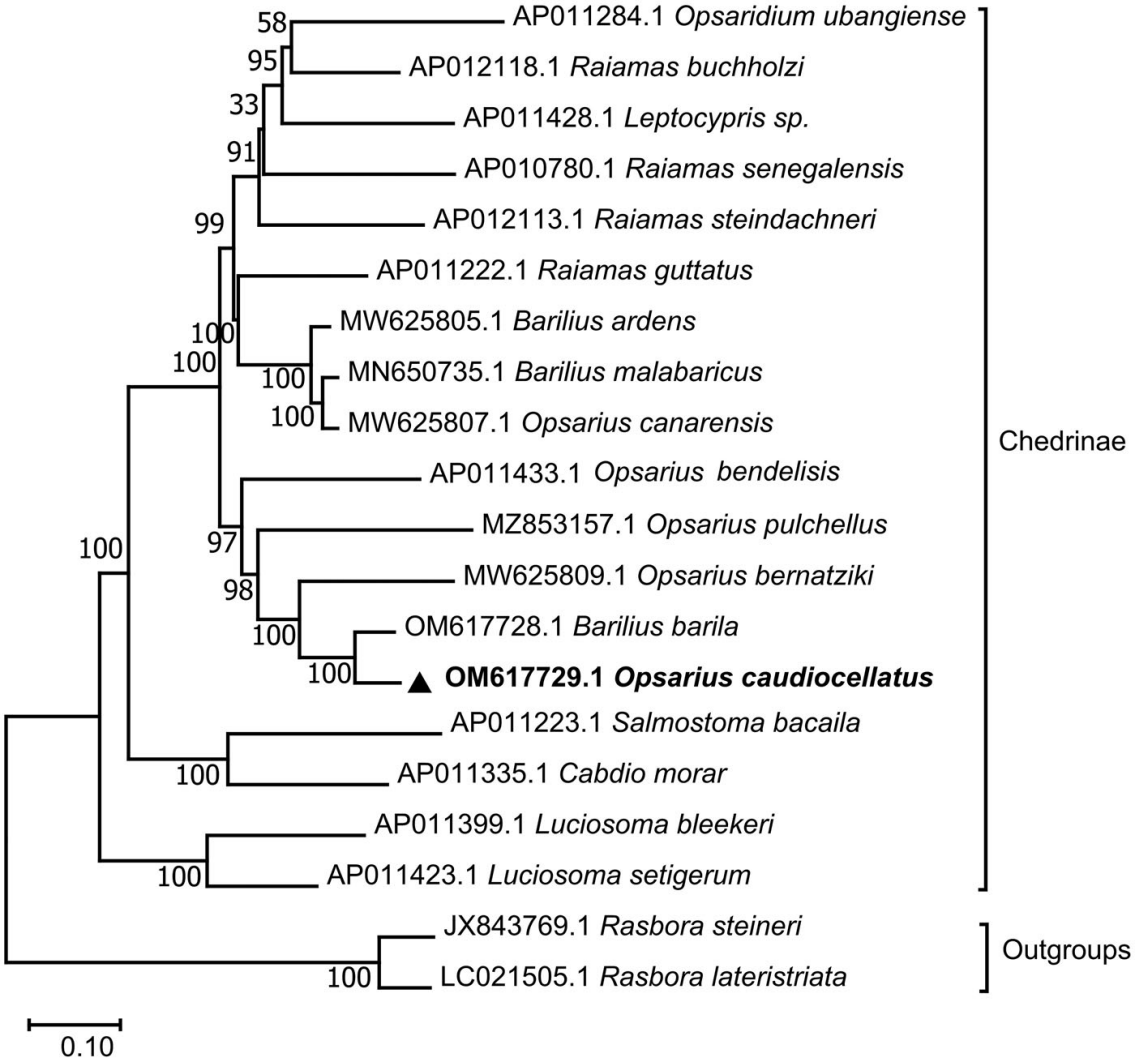
Maximum-likelihood (ML) phylogenetic tree was reconstructed using the concatenated mitochondrial protein-coding genes of *O. caudiocellatus* and other 19 fish species. Accession numbers were given next to the species’ names. The tree topology was evaluated by 1000 bootstrap replicates. Bootstrap values at the nodes correspond to the support values for ML methods.

The phylogenetic position of *O. caudiocellatus* in subfamily Chedrinae was shown in Figure 3. The phylogeny demonstrated that *O. caudiocellatus* clustered together with *Barilius barila, Opsarius bernatziki* (Yu et al. 2022)*, Opsarius pulchellus* (Chen et al. 2022), and *Opsarius bendelisis* (Tang et al. 2010) to form a sister group of the other clade clustered by *Opsaridium ubangiense* (Miya et al. 2016), *Raiamas buchholzi* (Miya et al. 2016), *Leptocypris sp.* (Tang et al. 2010), *Raiamas senegalensis* (Saitoh et al. 2011), *Raiamas steindachneri* (Miya et al. 2016), *Raiamas guttatus* (Miya et al. 2016), *Barilius ardens*, *Barilius malabaricus* (Prabhu et al. 2020), and *Opsarius canarensis*. Further, *O. caudiocellatus* was closely genetically related to *Barilius barila.* Howes (1980) divided *Barilius* (sensu lato) into *Barilius* and *Opsarius* (Howes 1980). Based on morphology (Tang et al. 2010) and molecular phylogeny (Liao et al. 2011), Qin et al. proposed that the genus *Barilius* from South Asia should be changed to *Opsarius* (Qin et al. 2019), so the *Barilius canarensis* be changed to *Opsarius canarensis*. Phylogenetic tree in this paper shows that *Barilius* and *Opsarius* are not monophyletic, and they gather in their respective branches. It is more reasonable that *Barilius barila* belongs to the genus Opsarius rather than *Barilius*, while *Opsarius canarensis* belongs to the genus *Barilius* rather than *Opsarius*.

## 4. Conclusions

The complete mitochondrial genome of *O. caudiocellatus* was sequenced and annotated *via* high-throughput sequencing technology. The assembly circular mitogenome was 16,534 bp long. The phylogenetic analysis results supported that *O. caudiocellatus* was clustered together with *B. barila*. It is suggested that *Barilius barila* belongs to *Opsarius*, while *Opsarius canarensis* belongs to *Barilius*. This requires more data for further analysis and confirmation. The fundamental genetic data presented here could be beneficial for further genetic and evolutionary researches on *O. caudiocellatus*.

## Supplementary Material

Supplemental Material

## Data Availability

The genome sequence data that support the findings of this study are openly available in GenBank of NCBI at (https://www.ncbi.nlm.nih.gov/) under the reference number OM617729. The associated ‘BioProject,’ ‘Bio-Sample’ and ‘SRA’ numbers are PRJNA808199, SAMN26036050, and SRR18066620 respectively.
