# Supplementary figures and images for: Phylogenetic relationship and characterization of the complete mitochondrial genome sequence of Opsarius caudiocellatus (Cypriniformes: Danionidae: Chedrinae)
Source: Mitochondrial DNA B Resour. 2022 Dec 7;7(12):2051–5. doi: 10.1080/23802359.2022.2151324 (PMC9744206; doi:10.1080/23802359.2022.2151324)

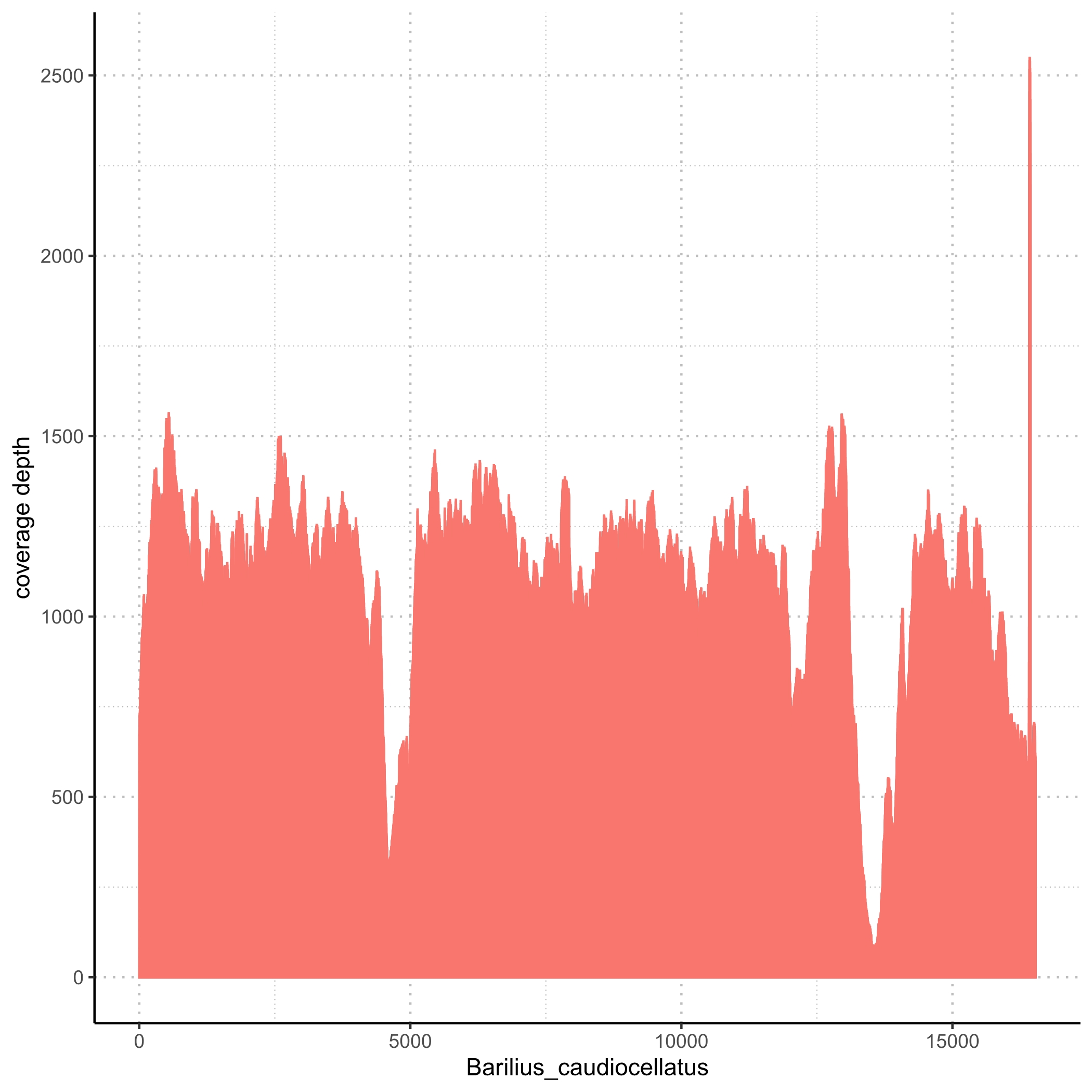

Supplement: Supplemental Material [file TMDN_A_2151324_SM3612.jpg]
